# Regulation of storage organ formation by long-distance tuberigen signals in potato

**DOI:** 10.1093/hr/uhae360

**Published:** 2025-01-03

**Authors:** Xinru Bao, Yunke Zhu, Guangcun Li, Lu Liu

**Affiliations:** Shanghai Collaborative Innovation Center of Agri-Seeds/Joint Center for Single Cell Biology, School of Agriculture and Biology, Shanghai Jiao Tong University, 800 Dongchuan Road, Minhang District, Shanghai 200240, China; Shanghai Collaborative Innovation Center of Agri-Seeds/Joint Center for Single Cell Biology, School of Agriculture and Biology, Shanghai Jiao Tong University, 800 Dongchuan Road, Minhang District, Shanghai 200240, China; State Key Laboratory of Vegetable Biobreeding, Key Laboratory of Biology and Genetic Improvement of Tuber and Root Crop of Ministry of Agriculture and Rural Affairs, Institute of Vegetables and Flowers, Chinese Academy of Agricultural Sciences, 12 Zhongguancun South Street, Haidian District, Beijing 100081, China; Shanghai Collaborative Innovation Center of Agri-Seeds/Joint Center for Single Cell Biology, School of Agriculture and Biology, Shanghai Jiao Tong University, 800 Dongchuan Road, Minhang District, Shanghai 200240, China

## Abstract

Potatoes are valued as reliable crops due to their high carbohydrate content and relatively low farming demands. Consequently, significant attention has been directed towards understanding and controlling the life cycle of potato tubers in recent years. Notably, recent studies have identified self-pruning 6A (StSP6A) as a key component of the tuberigen, the mobile signal for tuber formation, produced in leaves and then transported underground to induce tuber formation in potatoes. Recent progress in comprehending the signaling mechanisms that regulate StSP6A by photoperiod and ambient temperature components, its long-distance transport into underground tissue, and its involvement in regulating stolon tuberization has advanced significantly. Consequently, the modulation of StSP6A and other possible tuberigen signals, along with their regulatory pathways, significantly impacts potato domestication and crop yield. This progress highlights the differential regulation of tuberigen signals and their potential functions in promoting tuber formation.

## Introduction

Potatoes (*Solanum tuberosum*) are one of the most important crops globally, ranking among the top staple foods after wheat, rice, and maize. Renowned for their remarkable adaptability to diverse environmental conditions, potatoes have emerged as a dependable crop, flourishing in developed and developing regions [1]. Potatoes are also a good source of essential nutrients, including carbohydrates, vitamins, minerals, antioxidants, and dietary fiber [2]. This ubiquity, coupled with their nutritional richness, positions potatoes as a fundamental source of sustenance, contributing significantly to global food security. The closest ancestors of cultivated potatoes originate in the high-altitude areas of the Andes Mountains, within the Andes region. Indigenous communities in this region began cultivating potatoes at least 7000 years ago, fostering the development of a diverse array of potato varieties adapted to various altitudes and climates [1]. Over 10 000 potato varieties are now cultivated globally, spanning 130 countries [3].

Potato cultivation is highly sensitive to climate change, with rising temperatures, altered rainfall patterns, and extreme weather posing significant challenges [4–6]. Elevated temperatures can reduce tuber yields and quality, while drought stress hampers stolon elongation and nutrient allocation. Excessive rainfall increases the risk of diseases like late blight. These challenges highlight the need for climate-resilient potato cultivars and sustainable practices to ensure productivity and food security in a changing climate.

**Figure 1:**
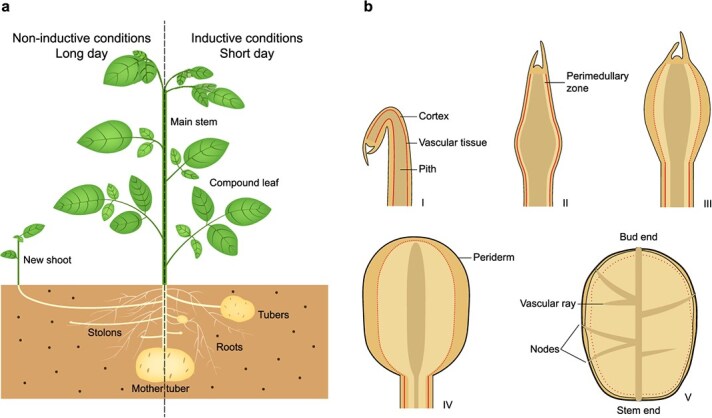
Diagrammatic illustration of potato growth and the developmental stages of potato tubers. **(a)** Under non-inductive conditions for tuberization (long days), potatoes prioritize vegetative growth. During this phase, stolons, specialized stems, elongate and may even grow vertically, emerging from the soil to give rise to new shoots. Under inductive conditions for tuberization (short days), potatoes undergo a significant shift in their growth patterns. Stolons undergo swelling and formation of tubers, which serve as storage organs for nutrients and energy. **(b)** Longitudinal sections of five stages (I–V) of potato tubers. Before swelling (I), a central pith and continuous vascular bundle are observed in the stolon tips [7]. At the onset of swelling (II), the central pith in the subapical region of the stolon tip enlarges, bending the vascular bundle around it [7]. As swelling progresses (III), the perimedullary region begins to thicken, with more pronounced vascular tearing, attributed to ongoing cell division that contributes to the bulk of the developing tuber [8]. In stage IV, the tuber starts to adopt its distinctive rounded shape, characterized by a thickened perimedullar region, the emergence of scattered vascular tissue, and the establishment of a robust periderm [8]. At the culmination of tuber maturation (V), vascular rays and nodes (eyes) are fully established, and the periderm hardens [9]. The tuber then enters a period of dormancy until the next growing season.

Potato plants, as a tuber-bearing crop, can reproduce sexually through seed production or asexually through tuber formation (Fig. 1a). Notably, the potato tuber is not formed from a root, but arises from an underground stem called a stolon that originates from the base of the plants and frequently emerges underground during the vegetative growth phase. Potato tubers store nutrients and energy and serve as a means of vegetative propagation. Potato tubers remain dormant in the soil during the cold winter and sprout out next spring to generate a new plant, thus serving as a strategy to bridge adverse conditions [10]. This tuberous vegetative propagation is also advantageous for plants as it allows plants to efficiently produce new offspring with the same genetic traits as the parents, ensuring the preservation of desirable characteristics.

This transition from stolon to tuber, known as tuberization, is a complex developmental process, which is orchestrated by intricate regulatory networks that respond to external environmental conditions and incorporate internal developmental signals. Day length (photoperiod) is the most reliable indicator of seasonal changes and is perceived by expanded leaves, which subsequently stimulate a mobile floral signal that moves long distances from leaves to the shoot apex to induce flowering. The molecular identity of this florigen remained elusive for nearly 70 years until multiple lines of evidence demonstrated that flowering locus T (FT) in Arabidopsis, along with its counterparts in other species, exerts a potent flowering-promoting effect and functions as a crucial component of the long-sought florigen [11–13]. *FT-*like genes have been identified in potatoes and were shown to undergo significant gene duplication, contributing to the emergence of novel functions involved in the regulation of tuber formation [14]. In this review, we will discuss the latest progress in understanding the regulation of *FT-*like genes in potatoes and illustrate how these tuberigen signals govern the potato stolon-to-tuber transition.

## Tuberization in potato

The ability of potatoes to produce tubers along the stolon is a crucial factor contributing to the success of asexual reproduction in potatoes. Under non-inductive conditions for tuberization, stolons often grow upward and emerge from the soil to form a new shoot. In contrast, in inductive growth conditions, stolons grow horizontally underground until the subapical region of the stolon swells to form the tuber (Fig. 1a). As tubers undergo growth and development, they progressively accumulate starch and other nutrients [8, 10].

Tuber morphology is determined by both cell division and cell enlargement. In the apical region of stolons, cells undergo transverse cell division and elongation, ensuring the growth of underground stolons (Fig. 1b). Upon tuberization, elongation of the stolon stops, and cells in the pith and cortex of the stolon subapical region undergo longitudinal division and enlargement, leading to the enlargement of the stolon tips. With further increase in size, longitudinal division ceases, and instead, random division occurs within the perimedullary region. This random orientation of cell division is accompanied by cell enlargement, contributing to the maturation of the tuber and ultimately leading to an increase in its final diameter [8, 15]. As the perimedullary zone thickens, the vascular tissue becomes irregularly arranged, and the tuber vascular pattern gradually forms [8]. All these processes are essential for the development of mature tubers, as they allow for the expansion and filling out of the tuber, resulting in the characteristic size and shape associated with fully grown potatoes.

One noteworthy change in tuberization is the shift in the sucrose unloading pathway in the phloem: from apoplastic to symplastic transport. Apoplastic phloem unloading predominates during stolon development. However, following tuberization, sucrose unloading from the phloem undergoes a transition from apoplastic to symplastic transport. This transition is crucial as symplastic gating prevents sucrose from passively leaking out of companion cells (CC) and parenchyma cells (PC) into the apoplast, thereby regulating sucrose transport within the tuber in a symplastic manner. Thus, this regulation is vital for effective resource storage [16, 17]. In addition, this transition is accompanied by significant alterations in enzyme activities and cellular differentiation. Specifically, there is a decrease in cell wall invertase activity and an increase in sucrose synthase activity in the subapical region of swelling stolons, along with the differentiation of proplastids into amyloplasts [16, 18, 19]. Together, these alterations facilitate the accumulation of starch in the storage organs.

## Photoperiodic control of tuberization signal in potato

The regular and predictable fluctuations in day length throughout the year serve as reliable indicators of changing seasons. Potatoes utilize these day-length signals to predict the upcoming environmental shifts, ensuring the timely formation of dormancy tubers before winter. The Andigena species (*S. tuberosum* ssp. *andigena*) strictly require short-day (SD) photoperiod for tuber formation. This species does not form tubers under long-day (LD) conditions or when the long night is interrupted by a pulse of light (night break) [20, 21]. Although modern potato genotypes are more adapted to LD conditions, tuberization in these cultivars is still promoted by reduced day lengths [2, 22].

Tuberization bears a strong resemblance to floral transition. In flowering plants, the leaves perceive the photoperiodic signal and generate a floral stimulus that traverses from the leaves to the shoot apex, ultimately triggering the flowering process. Graft experiments have evidenced the transmission of this floral stimulus from a flowering donor to a nonflowering receptor through the graft union [23]. Similarly, potato leaves generate signals that travel to the underground stolon, inducing tuberization in response to photoperiodic changes. Grafting leaves from potato plants cultivated under tuber-inductive conditions promotes tuberization in noninduced stocks. Moreover, grafting leaves from tobacco plants induced to flower leads to tuberization in potatoes grown under non-inductive conditions. Conversely, grafted leaves from noninductive conditions do not induce tuberization [20, 21]. These findings suggest that florigen, promoting flowering, and tuberigen, inducing tuberization, may represent universal and interchangeable signals.

**Figure 2:**
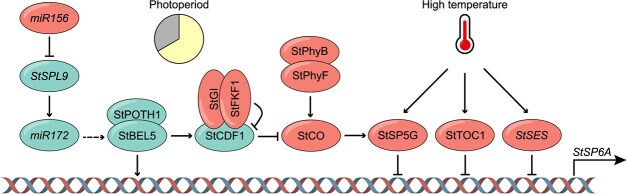
Transcriptional regulation of *StSP6A* in response to photoperiod and high temperature in potatoes. In the photoperiod pathway, two modules regulate the expression of *StSP6A*. (i) *miR156*-*StSPL9*-*miR172*-*StBEL5* module. *miR156* downregulates *miR172* abundance via *StSPL9* [28]. Reduced *miR172* levels are linked to decreased *StBEL5* mRNA, potentially through *RAP1* [29]. StBEL5, in conjunction with POTH1, activates the expression of *StCDF1* and *StSP6A* [30, 31]. The expression of *StSP6A* is suppressed by *miR156* and promoted by *miR172* [28, 29]. (ii) StCO-StSP5G module. Under LD conditions, the expression of *StSP6A* is suppressed by a regulatory cascade involving StCO and StSP5G. This suppression is facilitated by the stabilization of StCO protein, a process regulated by phytochromes PhyB and PhyF [32, 33], and the regulation of *StCO* transcription, which is mediated through the proteolytic degradation of StCDF1 protein regulated by StFKF1-StGI complex [34]. Under SD conditions, the evasion of StCDF1 from StFKF1/StGI-mediated degradation and the elevated expression level of *StCDF1* induced by StBEL5-POTH1 results in reduced *StCO* expression [30, 34]. This decrease in *StCO* expression indirectly promotes the activation of *StSP6A*. At high temperatures, the induction of *StSP5G*, *StTOC1*, and the pre-miRNA *StSES* contributes to the downregulation of *StSP6A*[35–37].

The discovery that ectopic expression of a rice *FT-*like gene *heading date 3A* (*Hd3a*) in leaves, which belongs to the family of phosphatidylethanolamine-binding proteins (PEBPs), induces tuberization in Andigena plants under non-inductive long days, suggests that *FT-*like genes could be key components of mobile tuberigen [24]. Moreover, studies on *FT-*like genes in onions (*Allium cepa* L.) and orchids *Dendrobium* Chao Praya Smile demonstrate their involvement in regulating both flowering and bulb formation [25, 26], illustrating the evolutionary conserved role of *FT-*like genes in promoting the development of storage organs. Sequencing of the potato genome identified 15 *FT*-like genes distributed across the FT-like, TFL1-like, and MFT-like clades. Among these, six genes belong specifically to the FT-like clades, including *self-pruning 6A* (*StSP6A*), *self-pruning 5G-A/B* (*StSP5G-A/B*), *StSP5G-*like *1/2*, and self-pruning *3D* (*StSP3D*) [14]. StSP6A is a master regulator of tuberization, with its expression strongly associated with tuberization, particularly in leaves and stolons of inductive plants. Suppression of *StSP6A* significantly delays tuber formation in SD conditions, and overexpression of *StSP6A* induces tuberization even in non-inductive LD conditions [24]. Therefore, unraveling the molecular mechanisms governing the regulation of StSP6A and understanding its role in promoting tuberization are crucial steps toward comprehending the regulation of potato tuber formation.

## Molecular mechanisms underlying regulation of StSP6A in leaves

### Regulation of StSP6A in response to changing day length

Arabidopsis is a facultative LD plant that flowers earlier under LD conditions. Transcriptional activation of *FT* occurs only under LD conditions and is closely associated with flowering. The day length-dependent activation of *FT* expression largely relies on constans (CO), which encodes a zinc-finger protein and activates *FT* expression by directly associated with *FT* promoter [11, 20, 27]. The activation of *FT* under LD conditions is tightly regulated by several transcriptional and post-translational mechanisms that control CO activity, ensuring its expression in the late afternoon [11].

In potatoes, the StCO-StSP6A pathway also defines the central module for tuber formation (Fig. 2). The ectopic expression of Arabidopsis CO in Andigena plants required an extended period to initiate tuber formation under SDs [38]. Similarly, overexpression of potato *StCO* delays tuberization under SD conditions, indicating that StCO functions as a repressor in potatoes [39]. In *StCO* silencing lines, tuberization occurs in non-inductive LD conditions, accompanied by upregulated *StSP6A* expression, while exhibiting no effect under strongly inductive SD conditions, suggesting that StCO regulates tuberization in a photoperiod-dependent manner [39].

The expression of *StCO* is tightly regulated by photoperiod. Under LD conditions, the expression of *StCO* exhibits a daily diurnal oscillation pattern, with its peak occurring relatively at dawn. While in SD conditions, the *StCO* oscillation pattern is altered, peaking 3 hours earlier than in LD conditions [32, 38, 39]. In Arabidopsis, the transcriptional regulation of *CO*, dependent on day length, is orchestrated by the cycling DOF factor (CDF) family protein and the flavin-binding, KELCH REPEAT, F-BOX 1 (FKF1)-GIGANTEA (GI) complex [40, 41]. FKF1 interacts with GI in a blue light-dependent manner and targets CDF1 proteins for degradation by the proteasome, thus releasing the repression of *CO* [42]. In potatoes, homologs of StCDF1 have been identified as crucial loci for LD acclimation in the tuberization process of a European potato type [34]. The early maturing descendant possesses three truncated alleles (StCDF1.2, StCDF1.3, and StCDF1.4) in addition to the full-length StCDF1.1. Unlike StCDF1.1, these three truncated alleles are stable and remain constant throughout the day, thereby repressing the expression of *StCO* and resulting in an early tuberization phenotype [34, 43].

The post-translational regulation of the CO protein by light also plays a crucial role in the day-length adaption of potatoes. StCO proteins are preferably accumulated to high levels in LDs but are significantly reduced in inductive SDs, since StCO protein is stable in light but undergoes degradation in darkness. Notably, this degradation occurs in far-red light but not in white, blue, or red light, indicating the involvement of cryptochrome (CRY) and phytochrome (PHY) photoreceptors in regulating StCO protein stability [32]. This is different from Arabidopsis CO, which accumulates in far-red light but is destabilized in red light, suggesting the evolution of different functions for photoreceptors in potatoes [11]. Consistently, phytochrome B (PhyB) has been identified to play a role in the photoperiodic control of tuber formation in potatoes. Potatoes with reduced levels of *StPhyB* lose control of tuberization by photoperiod and initiate tuberization very early under both LD and SD conditions. In *StPhyB* RNA interference (RNAi) lines, sessile tubers are formed directly attached to the main stem, indicating strong tuber induction signaling in these plants [20, 44]. Additionally, the tuber induction pathway is constitutively activated in *StPhyB*-RNAi lines, evidenced by a significant reduction in StCO protein levels in both LD and SD conditions, suggesting that the photoperiodic control of StPhyB through the regulation of StCO [32].

StSP6A plays a crucial role in inducing tuberization, exhibiting robust upregulation in SD conditions [24, 45]. Conversely, another FT homologue *StSP5G* is prominently abundant in leaves under LD conditions but diminishes to basal level in SDs [32]. Moreover, the elevated expression of *StSP5G* in LDs is significantly suppressed in *35S:StCDF1.2*, implying a role as a tuberization repressor for this *FT-*like homolog [34]. In *StSP5G*-RNAi lines, tuber induction occurs in LDs, and the tuber yield in SD conditions also sees an increase. Notably, the activation of *StSP6A* expression in leaves under LD conditions in *StSP5G*-RNAi lines suggests that StSP5G functions as a suppressor of *StSP6A*, thereby exerting control over tuberization [32]. The elevated expression of *StSP5G* shows a positive correlation with the accumulation of StCO protein, and the expression of *StSP5G* experiences a substantial downregulation in *StCO*-RNAi lines. This implies a direct targeting of *StSP5G* by StCO. Additional supporting evidence establishes that StCO binds to a conserved TGTGGT motif in the *StSP5G* promoter under LD conditions, thereby activating the expression of *StSP5G* [32]. Furthermore, *SlSP5G* in wild species of tomato is specifically expressed in LD. It is the major locus contributing to day-length adaption, suggesting that, as relatives, tomatoes and potatoes may have evolved similar mechanisms for day-length adaptation [46, 47].

Phytochromes serve as crucial photoreceptors responsible for perceiving, interpreting, and transmitting light signals [48]. Beyond *StPhyB*, *StPhyF* also plays a pivotal role in the photoperiodic regulation of tuberization in potatoes. The suppression of *StPhyF* results in tuber formation even under LD conditions. In the *StPhyF*-RNAi lines, the degradation of the StCO protein occurs, subsequently leading to the upregulation of *StSP6A* expression. Notably, StPhyF interacts with StPhyB to form a heterodimer, indicating that StPhyF and StPhyB operate within the same pathway to modulate the StCO-StSP6A pathway [33].

BEL-like transcription factors play a crucial role in plant morphogenesis and development. In potatoes, bellringer-1 like 5 (StBEL5) binds to numerous tuberization genes, including *StSP6A*, and regulates the onset of tuber formation [30, 31]. In plants with elevated levels of *StBEL5* and in *StBEL5*-RNAi lines, changes in *StSP6A* transcript levels are correlated with StBEL5 activity, suggesting that StBEL5 regulates *StSP6A*. Additionally, tandem TGAC motifs, recognized by BEL-like transcription factors, are identified in the promoter of *StSP6A* [49]. Mutations of these TGAC motifs in the *StSP6A* promoter significantly repress the SD-induced gene expression in leaves and stolons, indicating direct regulation of *StSP6A* by StBEL5 [30]. Notably, downstream genes activated by StBEL5 are also induced by StSP6A, demonstrating that the signaling pathways regulated by StBEL5 and StSP6A partially overlap [30]. StBEL5 also induces *StCDF1*, which contains six tandem TGAC motifs in the promoter region of *StCDF1* and, in turn, regulates the expression of *StSP6A* through a regulatory cascade involving StCO and StSP5G [30, 32, 50].

### Regulation of StSP6A in response to high temperature

Ambient temperature plays a pivotal role in governing diverse facets of plant development, with a notable impact on tuberization in potatoes. Elevated temperatures adversely influence tuberization, particularly during critical developmental phases, disrupting the intricate signaling and hormonal balance crucial for tuber initiation [51]. Under high temperatures, there is an augmented allocation of carbon sources to leaves and stems, accompanied by a decreased translocation of photoassimilates to tubers [52]. This alteration in the distribution of photoassimilates, diverting resources from tubers to vegetative tissues, signifies the plant’s prioritization of vegetative growth over tuberization, ultimately resulting in diminished tuber formation [35, 53, 54].

In Arabidopsis, the thermosensory pathway orchestrates a signal cascade that intricately influences the expression of FT through diverse regulatory mechanisms [55–59]. The heat-induced inhibition of tuber growth in potatoes also correlates with the decreased expression of *StSP6A* in both leaves and tubers (Fig. 2) [35, 37, 60]. Notably, elevated temperatures induce the expression of *StSP5G* in leaves, indicating that StSP5G also integrates ambient temperature signals to repress the expression of *StSP6A* [35]. Additionally, *timing OF CAB expression 1* (*StTOC1*), the potato homolog of the Arabidopsis core circadian clock gene *TOC1*, functions as a thermo-responsive transcriptional factor, assimilating ambient temperature signals to further repress the expression of *StSP6A* [36].

FT orthologs in other species, like *Brachypodium distachyon*, have been shown to undergo post-transcriptional regulation mediated by Pooideae-specific miR5200 [61], emphasizing the significance of post-transcriptional control of *FT-*like gene expression. Post-transcriptional regulation is also part of the regulatory mechanisms of *StSP6A*, as evidenced by the fact that constitutive overexpression of *StSP6A* in potatoes does not fully overcome the effect of high temperature on tuber development [54]. *StSP6A* is post-transcriptional repressed by a pre-miRNA *suppressing expression of SP6A* (*SES*) (Fig. 2). The expression of *SES* undergoes developmental regulation, gradually decreasing during the tuberization process, thereby alleviating the repression on *StSP6A* and allowing the upregulation of *StSP6A* expression. In response to elevated temperatures, *SES* transcripts are prominently upregulated, contributing to the heat-mediated downregulation of *StSP6A* expression. Consequently, SES functions as a modulator, fine-tuning the transition from the vegetative to the generative stage and influencing the source-to-sink balance in response to temperature fluctuations [37]. Consistently, the codon-optimized *StSP6A* to avoid the silence effect of *SES*, referred to as *StSP6A^cop^*, markedly enhances the expression strength of *StSP6A*. Furthermore, overexpression of *StSP6A^cop^* notably accelerates tuberization, augments tuber growth, and significantly improves the harvest index, underscoring the pivotal role of StSP6A in maintaining high sink strength [37].

**Figure 3:**
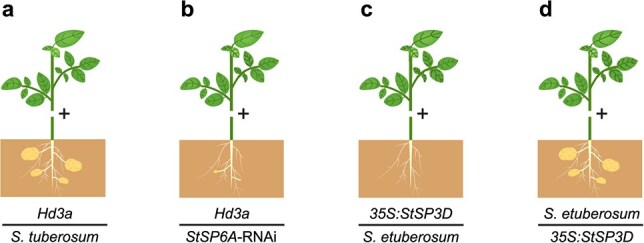
Diagrammatic illustration of tuberization in grafting combinations between tuberizing and/or non-tuberizing genotypes. **(a)** potato scions expressed with rice florigen *Hd3a* promote the tuber formation of *Solanum etuberosum* stocks [24]. **(b)** A delayed onset of tuberization is observed when grafting to *StSP6A*-RNAi stocks [24]. **(c)**  *S. etuberosum*, a non-tuber-bearing species, does not produce tubers when grafted onto *35S:StSP3D* scions [45, 64]. **(d)** Grafting *S. etuberosum* scions onto *35S:StSP3D* stocks result in tuber formation [45, 64]

*StSP6A* transcripts are regulated by ambient temperature via both transcriptional and post-transcriptional regulations at different growth stages of potatoes [54]. Examining the time course of *StSP6A* expression in leaves under elevated temperatures indicates an initial downregulation of *StSP6A* transcripts during the early stages of potato development. Intriguingly, at later stages, the overexpression of *StSP6A* reinstates the repression effect in the high-temperature conditions, indicating that *StSP6A* transcripts are post-transcriptional and transcriptional regulated at early stages but are mainly transcriptional regulated by late stages of potato growth [54].

The air and underground tissues of potatoes commonly experience distinct temperature conditions. Investigations into potatoes cultivated under combinations of different air and soil temperatures reveal that increased soil temperature substantially hampers tuber development. When potatoes are grown in warm soil, stolons tend to continuously elongate and frequently emerge from the soil, occasionally triggering tuberization upon encountering cooled air [62]. This observation implies that elevated soil temperatures impede the typical tuberization process. Moreover, when only the underground tissue of potatoes is heated, the expression of *StSP6A* in leaves is also affected, suggesting that long-distance signals transmit from the roots to the stolons and influence tuberization signals [60].

## Other tuberigen signals moving from leaves to stolons

Grafting experiments provide compelling evidence suggesting that FT-like proteins produced in leaves serve as tuber-inducing signals and are transported to underground stolons to initiate tuber formation [24, 63]. In addition to the primary tuber-inducing signal *StSP6A*, two additional florigen genes, *StSP3D* and *flowering locus T-like 1* (*StFTL1*), are also activated under SD conditions [24, 45]. Overexpression of *StSP3D* and *StFTL1* promotes tuber formation even in non-inductive LDs, and these two tuber induction signals are also graft transmissible, moving from leaves to stolons for tuber induction [45].

*Solanum etuberosum*, the sister species to the common ancestor of tomato and potato, cannot produce tubers. In this species, the fourth exon of *SeSP6A* is deleted, leading to a premature termination codon and an impaired phosphatidylethanolamine-binding protein domain. As a result, the expression of *SeSP6A* in *S. etuberosum* leaves is barely detected under both LD and SD conditions [64]. Grafting experiments using *S. etuberosum* as scions onto tuberizing potato types demonstrate that *S. etuberosum* can produce a tuberigen signal in its leaves, which promotes tuber formation in tuberizing-type scions (Fig. 3), suggesting that SP6A is not the only tuberigen signal generated in leaves [45, 65]. The signal generated in *S. etuberosum* for promoting tuberization is likely SeSP3D, as evidenced by the significantly delayed induction of tuber formation in *SeSP3D* knockout homozygous *S. etuberosum* grafts [45]. Furthermore, grafting experiments between tuberizing potato types and *S. etuberosum* reveal that, despite receiving the tuberigen signal from leaves, the *S. etuberosum* rootstock cannot induce tuber formation (Fig. 3) [45]. Notably, when grafting tuberizing potato types onto *StSP6A*-RNAi rootstock, the onset of tuberization is also substantially delayed [24]. These findings indicate that although StSP6A is a critical regulator promoting tuber formation in stolons, it is not the only tuberigen signal produced in leaves.

BEL-like transcription factors play a pivotal role in shaping plant morphology through their interactions with KNOTTED1-like homeobox (KNOX) proteins [66]. In potatoes, the onset of SD conditions triggers the accumulation of *StBEL5* transcripts in stolons. Subsequently, *StBEL5* transcript serves as a graft-transmissible signal, with its movement to the tuber intricately linked to enhanced tuber production [67]. This movement is induced by light and facilitated by two RNA-binding proteins, polypyrimidine tract-binding protein 1 (StPTB1) and StPTB6, which bind to the untranslated region of *StBEL5* mRNA [67–70]. Within stolons, StBEL5 collaborates with its protein partner, the knotted1-type transcription factor potato homeobox1 (POTH1), to regulate tuber formation [49, 67, 70, 71]. RNA sequencing reveals that the StBEL5-POTH1 complex induces the expression of tuber formation-related genes, notably *StSP6A* and *GA2ox1*, through binding to the tandem TTGAC motifs in the upstream sequences of these tuberization marker genes. Mutation of the putative binding site in the *StSP6A* and *GA2ox1* promoter significantly impairs the gene expression both in leaves and stolons, indicating that StBEL5 directly activates *StSP6A* and other tuberization genes [30, 49, 71]. Furthermore, the broader regulatory network involving StBEL5 in stolon tuberization awaits a comprehensive understanding.

Expanding the scope, two microRNAs, *miR172* and *miR156,* have been identified as key regulators of potato tuberization [28, 29]. In Arabidopsis, *miR172* and *miR156* control phase transition and flowering time. *miR172* facilitates the transition to the adult phase and promotes flowering, whereas *miR156* is crucial for juvenile stage development [72]. In potatoes, *miR172* promotes tuberization, and overexpression lines tuberize under non-inductive LD conditions. *miR172* is detectable in the vasculature of potato plants, and *35S:miR172* scions grafted onto wild-type stocks tuberize as early as *35S:miR172*/*35S:miR172* control, suggesting that *miR172* is a graft-transmissible signal or may regulate a long-distance tuberization signal [29]. *miR172* targets the *AP2-like* gene *related to apetala2 1* (*RAP1*) for degradation, so it was hypothesized that *RAP1* acts downstream of *miR172* to control tuber formation. Furthermore, the transcript level of *StBEL5* is upregulated in *35S:miR172*, possibly through RAP1. The expression of *miR172* is higher in all organs under SD conditions compared to LD conditions. Conversely, the level of another microRNA *miR156* is decreased in stems and leaves but increased significantly in stolons under tuber-induction SD conditions. Overexpression of *miR156* affects multiple morphological traits, notably, with *35S:miR156* plants producing aerial tubers and fewer underground tubers, resulting in reduced tuber yield. This indicates that *miR156* plays a role in tuber development [28]. The target genes of *miR156* encode a plant-specific family of transcription factors known as *squamosa promoter binding-likes* (*SPLs*) [72]. Overexpression of *miR156*-resistant *SPL9* leads to the accumulation of *miR172*, demonstrating that the *miR156*-*SPL* module regulates *miR172* [28]. *miR156* is also detected in the vascular tissue and functions as a graft-transmissible signal in potatoes [28]. These microRNAs add another dimension to the intricate molecular interplay underlying the complex process of tuber development in potatoes.

## Regulation of tuberization in stolons

### Formation of TAC complex

In Arabidopsis, FT proteins traffic from leaves to the shoot apical meristem. Following its arrival at the shoot apex, FT interacts with a bZIP transcription factor FD and 14–3-3 proteins. Together, they establish a complex known as the florigen activation complex (FAC), which activates the floral meristem identity genes, thereby initiating flower formation [73, 74]. In potatoes, StSP6A similarly forms a tuberigen activation complex (TAC) with a potato FD-like protein StFDL1 and St14–3-3 s [75]. Suppression of *StFDL1* delays tuberization, suggesting that this TAC-like complex is essential for potato tuber formation [75]. StSP6A also interacts with StABI5-like 1 (StABL1), another bZIP transcription factor possibly related to the abscisic acid (ABA) signaling pathway, and forms an alternative TAC (aTAC) to promote tuberization [76]. Another TCP transcription factor StABL1 and StSP6A-associated TCP protein 1 (StAST1) interacts with both StSP6A and StABL1. These interactions attenuate the formation of the aTAC, thereby repressing tuberization [77].

In Arabidopsis, FD additionally engages in interaction with another PEBP gene family member, terminal flower 1 (TFL1). This interaction creates a competitive scenario with FT, resulting in the assembly of a TFL1-FD transcriptional inhibitory complex. This protein complex acts to suppress the expression of floral meristem identity genes, thereby regulating and repressing the initiation of floral development [78–81]. The potato TFL1 homolog protein centroradialis (StCEN) operates similarly to impede the assembly of the TAC complex. Diminished expression of *StCEN* stimulates tuber formation, while overexpression of *StCEN* retards this process, resulting in a reduction in tuber yield [82]. These findings indicate the conservation of regulatory mechanisms among *FT-*like genes in governing downstream signaling pathways involved in both tuberization and floral transition.

### Self-regulation of StSP6A in stolon

In Arabidopsis, the FT protein, rather than its transcript, undergoes trafficking from leaves to the shoot apex, leading to an undetectable *FT* transcript in the shoot apical meristem [83–86]. In potatoes, FT-like protein also efficiently transports from leaves to stolons; however, the expression of *StSP6A* in stolons experiences a significant increase during tuberization. Furthermore, for SD-induced plants, the induction of *StSP6A* in stolon is delayed compared to leaves, suggesting an autoregulatory loop for the transported StSP6A to induce the *StSP6A* expression in stolon [24]. Thus, unlike Arabidopsis, the regulation of *StSP6A* in stolons involves a relay mechanism that sustains the production of the inducing signal in stolons. This autoregulation of *StSP6A* is crucial for tuber formation, as demonstrated by the substantial reduction of *StSP6A* transcripts in stolons when grafting scions expressing a rice florigen *Hd3a* onto *StSP6A*-RNAi stocks. The onset of tuberization is also delayed in *StSP6A*-RNAi stocks [24]. Furthermore, grafting with *S. etuberosum* rootstock, which carries a compromised *StSP6A*, has also demonstrated ineffectiveness in triggering tuber formation (Fig. 3) [45].

Several regulatory mechanisms contribute to the autoregulation of *StSP6A*. The *APETALA1* (*AP1*)*-like* gene *StMADS1*, which is regulated by TAC, plays a crucial role in tuberization. The suppression of *StMADS1* has been shown to delay tuberization in plants [87]. Under SD conditions, both *StMADS1* and *StSP6A* exhibit diurnal patterns, with the peaking expressions at the end of the dark period. Additionally, the *StSP6A* transcript is downregulated in *StMADS1*-RNAi plants, indicating feedback regulation between *StSP6A* and *StMADS1* [87]. StCO acts as a repressor of *StSP6A* in leaves [32]. Consistent with this role, StCO also represses the autoregulation of *StSP6A* in stolons. The expression of *StSP6A* is largely repressed in stolons of *35S:StSP6A*/*35S:StCO* grafted plants compared with *35S:StSP6A*/wild type control [24]. However, it is worth noting that StCO is unstable in dark conditions [32]. Therefore, investigating the presence of StCO proteins in underground stolons and exploring the post-translational regulation of StCO in stolons present intriguing avenues for further research.

Accumulated FT proteins in leaves participate in the feedback regulation of their transcript level in Arabidopsis. In transgenic plants exhibiting ectopic expression of *FT* in phloem CC, native *FT* expression is downregulated, despite the high levels of total *FT* transcripts. This observation suggests that the excessive accumulation of FT protein in phloem CC can directly or indirectly reduce the expression of native *FT* mRNA [88]. Similarly, this feedback regulation of the *StSP6A* transcript is also present in potatoes. In the dormant tuber buds of *StSP6A^cop^* transgenic plants, the amount of the endogenous *StSP6A* transcript is decreased, indicating an auto-regulation of native *StSP6A* transcript in potatoes due to the substantially high level of *StSP6A* transcripts [37]. These findings suggest that maintaining *StSP6A* at an optimized level is critical for specific developmental processes in plants.

### Unraveling the role of StSP6A in tuber formation

**Figure 4:**
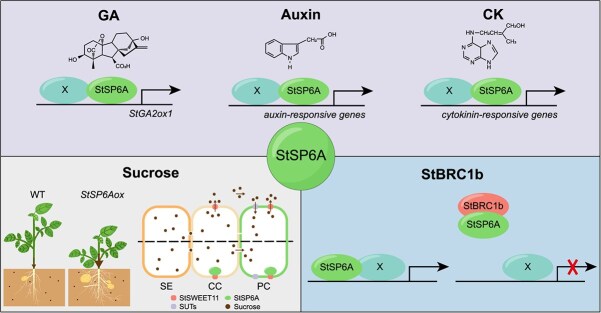
*StSP6A* regulatory pathways involved in stolon-to-tuber transition. Plant hormones (upper panel), sucrose (lower left panel), and StBRC1b (lower right panel) work in concert to regulate StSP6A. (upper panel) Plant hormones, including GA, auxin, and CK, play pivotal roles in the stolon-to-tuber transition. Elevated GA levels lead to delayed and reduced tuber formation. StSP6A induces the expression of *StGA2ox1* at the onset of tuberization, resulting in a localized decrease in active GA levels [89]. This decrease in active GA level facilitates the process of tuber formation. Auxin and CK have a promotive effect on the transition from stolon to tubers, and genes responsive to auxin and CK are also induced by StSP6A [24, 90, 91]. The allocation of sucrose to below-ground tissue is also important for tuberization. (lower left panel) Overexpression of *StSP6A* enhances the efficiency of sucrose transport to underground tissues [92]. Moreover, under noninductive conditions, sucrose can be unloaded through the sucrose efflux transporter StSWEET11 in CC and imported through the SUT and/or SUC H^+^-coupled sucrose symporters in phloem PC [93]. However, under inductive conditions, the interaction between StSP6A and StSWEET11 obstructs the passive leakage of sucrose to the apoplast of CC and PC, thereby shifting the mode of sucrose transport from the apoplastic to the symplastic pathway [93]. (lower right panel) In the aerial axillary buds, BRC1b interacts with and impairs StSP6A protein function, thus exerting a negative impact on above-ground tuberization [94]

The accumulation of the StSP6A signal in the stolon subapical region promotes the developmental switches in these group cells; however, the transcriptional regulators potentially acting downstream of TAC have yet to be identified. Various plant hormones play pivotal roles in stolon-to-tuber formation (Fig. 4). Gibberellin (GA), for instance, exhibits a negative impact on tuberization. Elevated GA levels lead to delayed and reduced tuberization [7]. A potato GA2-oxidase gene *StGA2ox1* is upregulated in the subapical region of the stolon at the onset of tuberization, causing a localized decrease in active GA levels and facilitating tuber formation [89]. Other GA oxidases, such as StGA3ox1 and StGA20ox, also play critical roles in modulating the bioactive GA pool, highlighting their importance in regulating tuber initiation and development [95, 96].

Another key phytohormone involved in tuber formation is auxin. The content of auxin in stolon tips increases during the early stages of tuber development, suggesting its promotive effects on tuberization. At the tuber swelling stage, GA levels sharply decrease, while auxin levels remain high and then slowly decrease [14, 97, 98]. Continuously applying auxin to stolon tips to maintain a high auxin level or overexpression of auxin biosynthesis *YUCCA* gene inhibits tuber formation, emphasizing the importance of an appropriate auxin peak in stolon swelling [40, 94, 99].

A transcriptome profiling of StSP6A-dependent tuber-specific genes reveals a substantial increase in *StGA2ox1* upon the induction of *StSP6A*. Simultaneously, several genes in the auxin signaling pathway, including *pin-formed* (*PIN1*), *PIN4*, and *auxin response factor 8* (*ARF8*), are transcriptionally induced [24, 90]. These findings suggest that StSP6A may directly target GA and auxin signaling pathways to promote the developmental transition.

Cytokinin (CK) is also recognized for its role in tuberization by facilitating cell proliferation and establishing a storage sink [100]. The promotive impact of exogenous CK on potato tuber development is evident from studies on in vitro-cultivated stem node cutting [7]. Conversely, diminishing the endogenous CK levels in potato plants through the expression of *cytokinin oxidase/dehydrogenase* (*CKX*) results in a decreased tuber yield [91]. Noteworthy is the observation that the ectopic expression of the tomato CK biosynthesis gene, *lonely guy 1* (*LOG1*), enables non-tuberizing tomato plants to form tuber-like organs in the axillary meristem. This suggests that CK might serve as a universal signal for the formation of storage organs [101]. In Arabidopsis, the FAC has been shown to regulate components in the CK pathway [81]. Consequently, exploring whether StSP6A also modulates CK signaling to induce tuber formation could be particularly interesting.

Brassinosteroids (BRs) are well-known regulators of cell elongation, division, and differentiation and have also been implicated in tuber formation. The BR receptor brassinosteroid-insensitive 1 (BRI1) interacts with plasma membrane (PM) proton ATPASE2 (PHA2), a proton pump localized to the plasma membrane. BRI1 phosphorylates PHA2, enhancing its activity and thereby promoting tuber development [102]. Optimal BR levels may synergize with other hormonal pathways to facilitate the developmental transition from stolons to tubers. However, the precise role of BR signaling in tuberization and its interplay with other hormones remain to be fully elucidated. Unraveling these mechanisms could provide valuable strategies for improving tuber yield and quality in potato crops.

Sucrose is another important signal for tuberization. A culture medium enriched with a high concentration of sucrose has been shown to effectively induce tuber formation from stem cutting, highlighting the role of sucrose in promoting and supporting the development of potato tubers [7]. Sucrose is synthesized in source leaves during photosynthesis and subsequently transported throughout the plant via the phloem system. Along this pathway, sucrose is continuously unloaded to support the growth of various plant organs [103, 104]. Various terminal sink tissues, including flowers, roots, and stolons, compete for the storage of the available sucrose. Altering the sucrose transporter and sucrose metabolism has been shown to regulate tuber production and starch accumulation [18, 105] Therefore, the efficiency of sucrose transport into underground stolons holds significance for starch accumulation in tubers.

StSP6A has been reported to be implicated in the regulation of sucrose allocation to below-ground tissues (Fig. 4) [92]. Overexpression of *StSP6A* results in reduced shoot growth but promotes root and lateral shoot outgrowth [37, 92], supporting the role of StSP6A in promoting belowground tissue growth. The use of the fluorescent sucrose analog esculin reveals the enhanced efficiency in sucrose transport to underground tissues in *StSP6A* overexpression lines [92]. Moreover, StSP6A engages with the sucrose efflux transporter sugars will eventually be exported transporter 11 (StSWEET11). This interaction inhibits the passive leakage of sucrose to the apoplast in phloem CC and PC, subsequently inducing sucrose symplastic transport into tuber parenchyma [93]. Notably, elevated sucrose levels also trigger an upregulation of *StSP6A* expression, further contributing to the promotion of source-sink partitioning [93].

An intriguing question arises: why is sucrose allocation favored towards belowground tissues in *35S:StSP6A* plants? A plausible explanation lies in the possibility that the StSP6A protein undergoes post-translational regulation or experiences modification in protein activity in different tissues. In potatoes, the *branched1b* (*StBRC1b*) encodes a teosinte branched 1/cycloidea/PCF (TCP) transcription factor that spatially regulates potato tuberization (Fig. 4) [94]. *StBRC1b* is expressed in aerial axillary buds and young stolons. Functioning as a tuberization repressor in aerial axillary buds, *StBRC1b*-RNAi plants show ectopic formation of aerial tubers but a reduction in underground tubers. StBRC1b interacts with StSP6A, aiming to inhibit its tuber-inducing activity. This interaction helps balance the sink strength of the aerial axillary meristem with stolons, ultimately promoting tuberization underground [94].

StBRC1b was also identified in a candidate gene screening for tuber development and was named *identity of tuber 1* (*IT1*) [64]. Using CRISPR-Cas9-based genome editing in diploid potatoes, IT1 was shown to be a key regulator of potato tuber initiation. In the *it1* mutant, stolons preferentially developed into branches rather than swelling at the sub-apical region, contrasting with the formation of aerial tubers and reduction in underground tubers observed in the *StBRC1b*-RNAi plants [64, 94]. This discrepancy raises an important question regarding the specific role of StBRC1b/IT1 in tuber formation. Several possible explanations exist: first, StBRC1b/IT1 may have distinct functions at different developmental stages, leading to phenotypic differences between knockdown and knockout mutants. Second, *StBRC1b*-RNAi plants and *it1* loss-of-function mutants were generated in different potato species, which may contribute to the variation in phenotypes. Additionally, tuber formation is influenced by multiple promoting factors, and the phenotypic differences in StBRC1b mutants may largely depend on the genetic background and ecotype. Further research is required to unravel the precise role of StBRC1b in tuber development.

Tuber formation in potatoes is a complex developmental process regulated by an intricate network of genetic and environmental factors. The efficient underground accumulation of carbon sources in potatoes can be better elucidated by isolating additional proteins that interact with StSP6A, providing a more comprehensive understanding of the intricate regulatory networks governing tuber development.

## Conclusion

In recent years, significant progress has been made in understanding the mechanisms underlying storage organ formation in potatoes. Key discoveries include the involvement of *FT-*like genes in controlling both flowering and storage organ development. StSP6A, a central regulator, integrates various developmental and environmental signals to control tuber formation in potatoes. Many upstream regulators of *StSP6A* have been identified, mutations of which can either promote or delay tuberization. StSP6A functions similarly to the florigen FT, moving long distances from leaves to underground tissues to induce tuberization. However, an autoregulatory mechanism induces high expression levels of *StSP6A* in stolons, which is critical for the transition from stolons to tubers. StSP6A forms a transcriptional complex with other coregulators in stolon tips, regulating hormone signaling pathways that control cell division and photoassimilate allocation, ensuring efficient sucrose transport into underground tissues. Despite these progresses, the regulatory mechanisms involved are still largely unknown.

The expression of *FT*-like gene in potatoes promotes tuber formation, and the overexpression of *StSP6A* in Arabidopsis induces early flowering [24], suggesting that these PEBP family proteins retain similar functions and can function interchangeably. This raises the intriguing question: why do proteins with similar functions promote different developmental processes? Understanding how potatoes translate this leaf-derived long-distance signals to induce the transition of underground stolons into tubers could provide valuable insights into the molecular identity of PEBP family proteins in plant development.

In potatoes, all axillary buds, including above-ground buds, have the potential to develop into tubers. Under tuber-inducing conditions, the expression of *StSP6A* is also induced in axillary buds [94], yet only the underground stolons transform into tubers. This suggests the unknown factors specifically located underground interact with StSP6A, triggering the tuber formation signaling cascades. StBRC1b/IT1 was identified as a key factor in transmitting the StSP6A signal for tuber induction. However, it was also found to inhibit aerial tuber formation in axillary buds [64, 94]. This intriguing dual role warrants further investigation to uncover the intricate function of StBRC1b/IT1 in tuber development. Furthermore, potato tubers are a form of vegetative propagation, a type of asexual reproduction. Like seed plants, the tubers store nutrients that support the initial growth of new plants. Seed development and storage product accumulation have been extensively studied in seed-producing plants [106–108], the regulatory pathways governing bioproduct storage are likely to be somewhat conserved. Understanding these pathways could offer valuable insights into the development of potato tubers.

Several florigen transport types of machinery have been identified in Arabidopsis and rice [55, 88, 109–112]. Similarly, the transport system delivering tuberigen signals, such as StSP6A, to the subapical region of stolons is critical for tuber formation, yet the mechanisms governing this process remain unclear. Future research should aim to identify the transporter and unloading mechanisms that facilitate StSP6A movement from leaves to stolons, providing key insights into the regulation of storage organ development and strategies to enhance crop yields.

Beyond tuber formation, StSP6A is implicated in the development of other storage organs in plants. Its role as a systemic signal for resource allocation suggests it may drive the initiation and growth of structures such as bulbs, rhizomes, or enlarged roots in various species [10, 13, 24, 113]. For instance, SP6A homologs in crops like onion or carrot might regulate analogous developmental pathways, coordinating carbohydrate allocation and storage organ formation. Exploring the broader functions of SP6A across species could reveal conserved mechanisms underlying storage development and provide opportunities to optimize storage organ development in diverse crops.

Cultivated potatoes have a diverse gene pool derived from the domestication of wild *Solanum* species, providing adaptability to various growing conditions [114]. Resequencing the genomes of diverse potato cultivars and wild species has identified numerous loci under selection, associated with traits such as early maturation, day-length and temperature adaption, disease resistance, and signature varieties [43, 64, 114, 115]. Furthermore, deleterious recessive alleles in potatoes affecting survival and growth vigor are retained in the potato genome due to long-term vegetative propagation. Whole-genome sequencing and evolutionary strategies have been employed to identify these deleterious mutations and key factors for potato breeding [64, 116, 117], enhancing our understanding of potato evolutionary adaption and laying the groundwork for genome-based breeding efforts.

## Data Availability

No application to this article as no datasets were generated or analyzed during the current study.
